# Nursing students’ attitudes and intentions towards seeking professional psychological help: the mediating role of emotional intelligence

**DOI:** 10.1186/s40359-025-02474-w

**Published:** 2025-02-20

**Authors:** Samirh Said Alqhtani, Nouf Afit Aldhafeeri, Lina Alotaibi, Huda Alanzi, Wafa Alshamrani, Norah Alqhtani, Abeer Selim

**Affiliations:** 1 College of Nursing, King Saud bin Abdulaziz University for Health Sciences, Riyadh, Saudi Arabia; 2 King Abdullah International Medical Research Center, Riyadh, Saudi Arabia; 3 Faculty of Nursing, Psychiatric and Mental Health Nursing Department, Mansoura University, Mansoura, Egypt

**Keywords:** Professional psychological help, Attitudes, Intentions, Emotional intelligence, Mental health, Nurse

## Abstract

**Background:**

It has been proposed that nursing students experience emotional and mental issues due to their educational demands, which influence their academic success. This study aims to explore the relationship between students’ attitudes and intentions toward seeking professional psychological help and focus on exploring a mediating role of emotional intelligence.

**Methods:**

This study used a descriptive correlational design. A convenience sample was used to recruit 514 undergraduate nursing students in the central region of Saudi Arabia. Data were collected using self-administered tools from three nursing colleges. Data were analyzed using both descriptive, inferential, and structural equation modeling statistics.

**Results:**

The current study results indicate that university students had moderate positive attitudes, moderate positive intentions toward seeking help for mental health concerns, and a positive level of emotional intelligence. Attitudes toward seeking help and emotional intelligence had a significant positive relationship on professional mental help-seeking intentions. Other factors that predict students’ intention to seek help include the history of consulting a mental health professional and students who were unsure of the availability of the on-campus counseling center (*p* <.001). Emotional intelligence has a significant mediation effect on the relationship between mental help-seeking intention and mental help-seeking attitudes toward professional psychological help.

**Conclusions:**

Based on the results, students with emotional intelligence and positive attitudes toward seeking mental help were significantly correlated to their intentions of seeking professional mental help. This study proposes the importance of implementing interventional programs to increase nursing students’ intentions to use the university counseling center for optimum mental health and well-being.

**Supplementary Information:**

The online version contains supplementary material available at 10.1186/s40359-025-02474-w.

## Background

Mental health issues among nursing students are a global problem. The World Health Organization (WHO) recognizes the importance of mental health related to health and education in achieving the Sustainable Development Goals (SDGs) [1]. The SDGs emphasize promoting mental health and well-being among students as a fundamental aspect of sustainable development [1]. Depression and anxiety symptoms are commonly reported among college students, affecting their quality of life and academic achievement [2, 3]. Nursing students face unique challenges due to the demanding nature of their training and professional environment, which may influence their help-seeking behaviors differently than other student populations [4, 5]. Evidence shows that students view seeking psychological treatment as unnecessary (66%), a lack of time (26.8%), and a preference for self-management (18%) as the primary obstacles to seeking help, with only 12% mentioning stigma [6]. The situation is further complicated for students by academic challenges in the university environment, where the demands are higher, there is a focus on specialized areas of study, and increased pressure for career readiness [7]. Due to these challenges, students delay seeking psychological help from professional or non-professional sources to effectively navigate these obstacles [8].

Help-seeking for mental health is a sequential process that must start with self-identification of symptoms, identifying these symptoms as indicative of a mental health concern, forming an intention, and ultimately leading to active engagement in help-seeking [9]. Professional help-seeking intention defines as the subjective chance of seeking help from mental health professionals [10]. Research indicates that positive attitudes toward help-seeking are related to both formal and informal sources of personal-emotional issues. Nursing students prefer disclosing their stress to informal support like family and friends over professional counseling [11]. Barriers to seeking professional help include normalizing symptoms and failing to recognize the problem [12]. Help-seeking behavior is a critical framework for exploring and understanding individual delays and prompt actions in response to various health conditions [13]. Factors influencing help-seeking behavior include stigma, lack of resources awareness, concerns about confidentiality, and perception of ineffective interventions [14, 15]. Social and self-stigmatization have a significant impact on individuals’ attitudes toward mental health treatment [16–18]. Demographic characteristics also influence help-seeking attitudes toward seeking psychological help. Female students exhibit more positive attitudes, while male students demonstrate higher levels of social and self-stigma [18–21]. Furthermore, positive views toward seeking help were more prevalent among female students who were aware of the on-campus psychological services [22].

The Theory of Planned Behavior (TPB) helps explain human health behaviors.

The TPB proposes that an individual’s intention to engage in a specific behavior and perceived control over its performance influence their decision to adopt that behavior [23]. Intentions reflect a person’s motivation or conscious plan to exert effort to perform the behavior [23]. The TPB stated that attitudes, perceived behavioral control, and subjective norms impact help seeking intentions [23]. Attitudes have been shown to predict help-seeking intentions [24]. A study reported the significance of attitude and perceived behavioral control in predicting intention to seek help and behavior among university students [25]. Low help-seeking intentions correspond to lower help-seeking behaviors [26]. Negative attitudes toward seeking professional psychological help present significant barriers to help-seeking [16, 17], while positive attitudes significantly impact individuals’ readiness to seek help [27, 28]. Moreover, in Saudi Arabia, a systematic review finding [29] showed that perceived barriers to seeking mental health services involve stigma, lack of awareness, and concerns about confidentiality, which may explain the gap observed in this study. In addition, in traditional culture, seeking psychological help might be perceived as a weakness. This cultural expectation may delay seeking psychological support and avoid showing vulnerability [18, 30].

Emotional intelligence (EI) plays critical roles for improving mental health and personal achievement. It includes intrapersonal and interpersonal skills that contribute to adaptive behavior and beneficial mental health outcomes [31]. Increased levels of EI are associated with enhanced mental well-being and better management of psychological challenges [32]. Researchers reported a positive correlation between EI and psychological help-seeking [33]. EI has been linked to factors that help prevent mental health problems, such as anxiety, stress, and depression [31]. Additionally, EI and psychological help-seeking behaviors positively correlate with academic achievement [34, 35]. Evidence suggests that EI can be developed over time, improving individuals’ capacity to seek and benefit from professional help [7].

The role of EI and help-seeking attitudes in predicting help-seeking intention is not explored, particularly among nursing students. Prior research has focused on the barriers and factors that influence the decision of individuals to seek professional psychological help. However, there is a need for research that considers the context of culture and its social and educational influences in Saudi Arabia, which play a significant role in mental health perceptions. The findings of this study offer a unique perspective on how nursing students’ EI and attitudes toward seeking professional help contribute to their mental health outcomes. This research can inform university administrators to implement interventions that improve EI and promote positive mental health behaviors, ultimately benefiting nursing students’ well-being and academic success.

### Aim of the study

This study aims to investigate the relationship between students’ attitudes and intentions toward seeking professional psychological help, with a focus on exploring the mediating role of emotional intelligence.

### Methodology

#### Design and participants

A descriptive correlational design was used to investigate the relationships between variables. The descriptive correlational design is best used to describe and investigate the association between study variables [36]. The convenience sample was used due to easy access to the target population, and it is an appropriate method for the current study design. Researchers recruited students who are enrolled in three nursing colleges in the central region of Saudi Arabia. Graduate students who have completed their undergraduate studies and are pursuing advanced degrees were excluded. The study sample was calculated using the G Power 3.1.9.7 program, with parameters including alpha = 0.05, medium effect size = 0.1, and predictors up to 32. The projected minimum sample size for the identified population was 424 students. However, considering the attrition rate, the sample size increased by 20%, making the final minimum sample size 509. The sample obtained was 558 nursing students; 55 were excluded due to missing data. Consequently, the final sample of 514 was included in the study. The study complied with the Strengthening the Reporting Requirements of Observational Studies in Epidemiology (STROBE) standards.

### Data collection

The data were collected from May to July 2024 using a self-reporting survey from nursing students at three public universities in Riyadh City, Saudi Arabia. The Qualtrics XM Platform was used and distributed via social media networks (Telegram and WhatsApp) to maximize the response rate. The online survey consisted of a cover page with an invitation to participate, as well as the aim of the study, followed by a consent form for participants who decided to participate, a demographic questionnaire, a Mental Help-Seeking Intention Scale, Mental Help-Seeking Attitude Scale, and Wong and Law Emotional Intelligence Scale (WLEIS). The estimated time for survey completion was 6–10 min.

### Measurements

The demographic data included age, gender, year of study, university name, history of consulting a mental health professional, history of using the on-campus counseling center, availability of the on-campus counseling center, and the source of knowing about on-campus counseling center. The Mental Help-Seeking Attitudes Scale (MHSAS) was used to assess mental health attitudes towards seeking help during times of distress from mental health professionals. This instrument consisted of 9 items, each utilizing a seven-point scale. A lower score indicates a negative attitude, and a higher score signifies a positive attitude [37]. MHSAS scale reliability is 0.95 [38]. The instrument exhibited high internal consistency (α = 0.92), and its test-retest reliability was confirmed by a significant bivariate correlation [38].

The Mental Help-seeking Intention Scale (MHSIS) was used to measure the students’ intention to seek help from professional. MHSIS is a 3-item scale that uses a 7 point rating scale. A lower score indicates a low intention to seek help, while a higher score indicates a stronger intention to seek help. The MHSIS has demonstrated evidence of internal consistency α = 0.94 [39] and α = 0.98 [40].

The Wong and Law Emotional Intelligence Scale (WLEIS) was used to measure the students’ EI. WLEIS is a 7-point Likert scale with 16 items divided into four subscales which are Self-Emotions Appraisal (SEA), Others-Emotions Appraisal (OEA), Use of Emotion (UOE), and Regulation of Emotion (ROE) [41]. The Cronbach’s alpha ranged from 0.80 to 0.89 [42]. The highest scores represent a higher level of emotional intelligence, and lower scores indicate less emotional intelligence.

### Reliability of the MHSAS, MHSIS, and WLEIS

In this study, the MHSAS measure revealed a very good level of internal consistency with a Cronbach’s alpha of 0.85 (95% CI: 0.83 to 0.87). Similarly, both MHSIS and WLEIS exhibited excellent internal consistency, achieving Cronbach’s alphas of 0.91, with 95% confidence intervals of 0.88 to 0.92 and 0.90 to 0.93, respectively.

### Data management and analysis plan

All analyses were conducted using R software version 4.2.2. Descriptive statistics were used to explain the participant characteristics and to determine the variable levels. Multiple regression analysis was used to determine predictors of the MHSIS. Mediation analysis using the Preacher & Hayes method was performed to test EI as a mediator. A significant level of 0.05 was used in all analyses.

## Results

### Participant characteristics

Females’ students represent the majority of the participants (81.3%), and the mean age was 20.60 years (SD = 1.62), with ages ranging from 19 to 31 years. Participants’ educational levels were distributed as follows: 11.9% were at first-year, 25.7% were at second-year, 32.1% were at third-year, 17.7% were at fourth year, and 12.6% were interns. A quarter of the participants (25.9%) reported a history of consulting a mental health professional. The availability of an on-campus counseling center or mental health clinic was confirmed by 75.3% of participants, whereas 19.5% reported no availability, and 5.3% were unsure. Additionally, 14.8% of the participants had utilized the on-campus counseling center or mental health clinic. Information about the on-campus counseling center was most commonly received from friends (31.3%), followed by academic advisors (28.8%), the university website (20.0%), the orientation program (11.7%), and other sources (11.1%) (Table 1).

**Table 1:** Participants characteristics

Variable	(*N* = 514)	%
Age, Mean ± SD, (Min-Max)	20.60 ± 1.62	(16–31)
Gender		
Female	418	81.3
Male	96	18.7
Year of study		
First	61	11.9
Second	132	25.7
Third	165	32.1
Fourth	91	17.7
Internship	65	12.6
University		
A	187	36.4
B	182	35.4
C	145	28.2
History of consulting a mental health professional	133	25.9
Availability of on-campus counseling center/ mental health clinic at your university		
Yes	387	75.3
No	100	19.5
I do not know	27	5.3
History of using the on-campus counseling center/ mental health clinic	76	14.8
From whom have the student heard about the on-campus counseling center		
Friends	161	31.3
Academic Advisor	148	28.8
The university Website	103	20.0
Orientation program	60	11.7
Other	57	11.1

### MHSAS and MHSIS

The overall MHSAS was (M = 5.13, SD = 1.20), indicating generally positive attitudes toward seeking help for mental health concerns. The items of MHSAS ranged from 5 to 6, with interquartile ranges (IQR) between 4 and 7. The overall MHSIS was (M = 4.45, SD = 1.78), reflecting moderate to positive intentions to seek help for mental health concerns. The MHSIS items ranged from 4 to 5, with an IQR between 3 and 7 (Table 2).

**Table 2:** MHSAS and MHSIS among the participating university students

Variable	Mean	SD
**MHSAS**	5.13	1.20
**MHSIS**	4.45	1.78

### WLEIS

The overall WLEIS was (M = 4.85, SD = 1.10), reflecting a moderate positive level of EI among the participants. For the subscale SEA was (M = 4.68, SD = 1.40), indicating a moderately positive self-emotional appraisal. For the OEA subscale was (M = 5.05, SD = 1.29), indicating a generally positive appraisal of others’ emotions. The UOE subscale was (M = 5.04, SD = 1.38), indicating a generally positive use of emotions. The ROE subscale was (M = 4.65, SD = 1.39), indicating a moderately positive regulation of emotions (Table 3).

**Table 3:** WLEIS among the participating university students

Variable	Mean	SD
SEA	4.68	1.40
OEA	5.05	1.29
UOE	5.04	1.38
ROE	4.65	1.39
**Overall WLEIS**	4.85	1.10

### Predictors of MHSIS

The multiple linear regression analysis examines the effects of the MHSAS and the WLEIS on the MHSIS while accounting for participants’ characteristics (Table 4). Significant predictors include the overall WLEIS, which had a coefficient of 0.32 (95% CI: 0.19 to 0.44, *p* <.001), indicating a significant positive effect on MHSIS. Similarly, the overall MHSAS showed a coefficient of 0.77 (95% CI: 0.65 to 0.88, *p* <.001), reflecting a significant positive effect on MHSIS. The history of consulting a mental health professional was another significant predictor, with a coefficient of 0.44 (95% CI: 0.14 to 0.74, *p* =.004), suggesting a positive impact on MHSIS. Additionally, the participants who said that no on-campus counseling center or mental health clinic was available compared to those unsure about its availability had a coefficient of 0.77 (95% CI: 0.16 to 1.37, *p* =.014), indicating a significant positive effect. Other characteristics, including age, gender, education level, university affiliation, and source of information about the on-campus counseling center, were not significant predictors of MHSIS.

**Table 4:** Multiple linear regression analysis results for examining the effects of MHSAS and WLEIS on MHSIS, accounting for participants characteristics

Predictors	ß	SE	95% CI		*p*
			Lower	Upper	
(Intercept)	-1.52	1.06	-3.60	0.56	0.152
Overall WLEIS	0.32	0.06	0.19	0.44	< 0.001*
Overall MHSAS	0.77	0.06	0.65	0.88	< 0.001*
Age	-0.01	0.05	-0.10	0.09	0.902
Gender (reference: female)					
Male	-0.19	0.21	-0.59	0.22	0.370
Education (reference: first year)					
Second	0.12	0.23	-0.34	0.57	0.612
Third	0.21	0.24	-0.25	0.67	0.377
Fourth	-0.03	0.27	-0.55	0.49	0.903
Internship	0.27	0.31	-0.34	0.88	0.384
University (reference: A)					
B	-0.04	0.18	-0.40	0.32	0.822
C	-0.17	0.17	-0.50	0.15	0.300
History of consulting a mental health professional	0.44	0.15	0.14	0.74	0.004*
Availability of on-campus counseling center/ mental health clinic at your university (Reference: I don’t know)					
No	0.77	0.31	0.16	1.37	0.014*
Yes	0.61	0.33	-0.03	1.25	0.064
Source of information about the on-campus counseling center					
Friends	-0.25	0.17	-0.58	0.09	0.148
Academic advisor	-0.06	0.16	-0.38	0.26	0.713
Orientation program	-0.36	0.21	-0.77	0.05	0.090
University website	0.03	0.18	-0.33	0.39	0.885
Other	-0.02	0.25	-0.51	0.48	0.951

### Mediation analyses for WLEIS on the effect of MHSAS on MHSIS

The mediation analyses for the effect of the MHSAS on MHSIS through the WLEIS were conducted in the mediation analysis using the Preacher & Hayes method with bootstrap [43] (2004). The researchers employed bootstrapping with 1000 samples to estimate the confidence intervals for the indirect effects of WLEIS on the relationship between MHSAS and MHSIS. The criteria for assessing the significance of the indirect effect was based on the 95% confidence intervals derived from these bootstrap samples.

The Average Causal Mediation Effect of WLEIS on the relationship between.

**Fig. 1:**
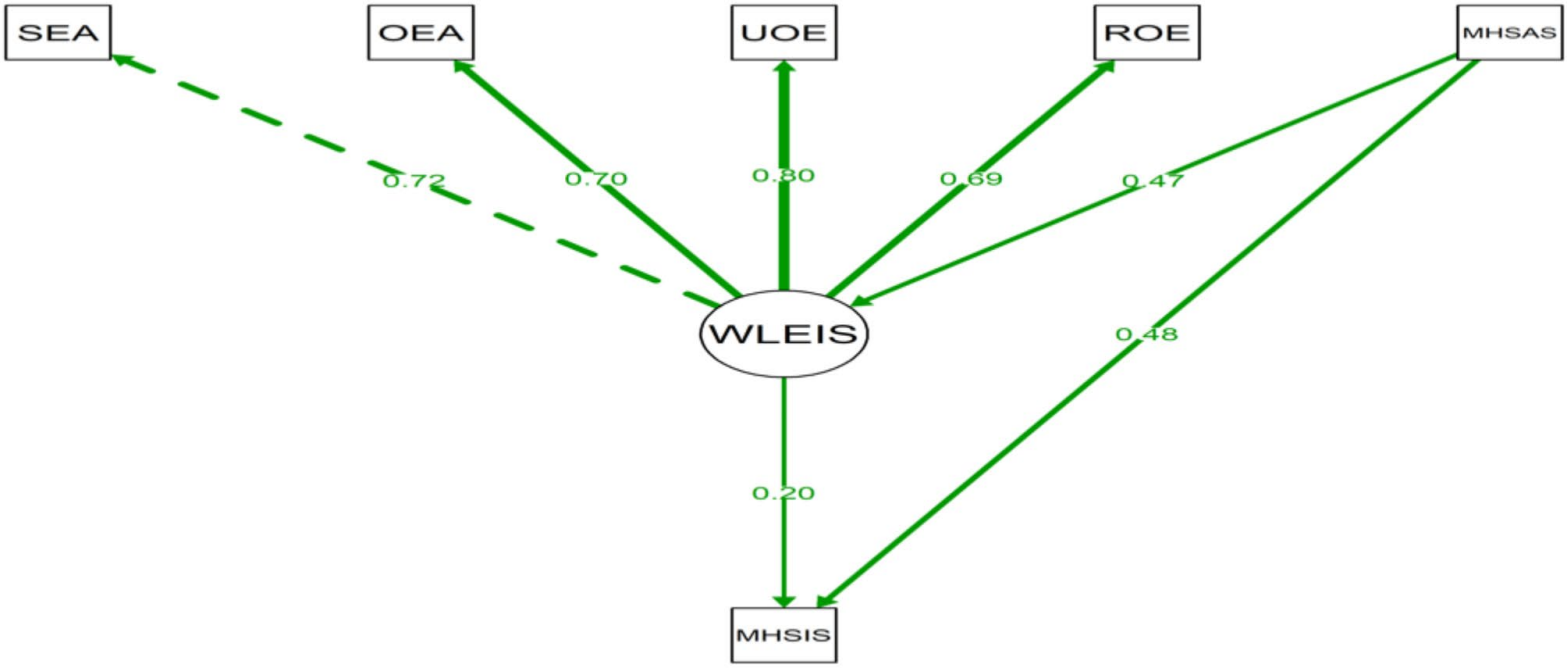
Path diagram showing the relationship between MHSAS, WLEIS, and MHSIS (outcome)

MHSAS and MHSIS was 0.11 (95% CI: 0.05 to 0.17, *p* <.001), indicating a significant mediation effect. The Average Direct Effect of MHSAS on MHSIS, including WLEIS in the model, was 0.75 (95% CI: 0.62 to 0.85, *p* <.001), indicating that WLEIS served as a partial mediator. While WLEIS accounts for some of the effect of MHSAS on MHSIS, MHSAS also independently influences MHSIS (Table 5). The Total Effect, combining both direct and mediated effects, was 0.86 (95% CI: 0.75 to 0.95, *p* <.001). The Proportion of Mediated Effect was 0.13 (95% CI: 0.06 to 0.20, *p* <.001), suggesting that approximately 13% of the total effect of MHSAS on MHSIS was mediated through WLEIS (Fig. 1).

**Table 5:** Mediation analyses for WLEIS on the effect of MHSAS on MHSIS

Effects	ß	95% CI		*p*
		Lowe	Upper	
Average Causal Mediation Effect (AB)	0.11	0.05	0.17	< 0.001*
Average Direct Effect (C)	0.75	0.62	0.85	< 0.001*
Total effect (AB + C)	0.86	0.75	0.95	< 0.001*
Proportion of Mediated effect	0.13	0.06	0.20	< 0.001*

## Discussion

This study provides valuable insights into nursing students’ help-seeking attitudes and EI in relation to their intentions of seeking psychological help. The demographic data of this study indicated that the majority of nursing students were female, with an average age of 20 years. This is consistent with other studies that show females dominate the population in nursing programs [44, 45]. In this study, a large proportion of participants are in their second and third years, suggesting increased academic stress throughout their studies. This might be due to nursing students’ transition from foundational, where courses are easier than specialized nursing courses and clinical training. These findings were consistent with [18, 46]. Therefore, this study reinforces the importance of tailored interventions that address students’ emotional and academic needs of nursing students during these critical stages of education.

In this study, most of the participants confirmed the presence of an on-campus counseling center or mental health clinic. Despite this, a smaller percentage (25.9%) of students had a history of consulting a mental health professional, showing that one in four students had visited mental health services. This result suggests a significant gap between service availability and utilization. This can be explained by the fact that young adults are concerned about exposing their emotional vulnerabilities and lack of trust in mental health professionals, which contributes to the barriers of seeking professional psychological help [47, 48]. Furthermore, the cultural barriers and stigma associated with visiting mental health centers have led to low utilization of these services [18]. From the researcher’s perspective, low utilization rates could be caused by a lack of awareness about how to access mental health services, long waiting times, or concerns about confidentiality.

The current study found that friends and academic advisors were the primary sources of information regarding the availability of psychological resources among students. This aligns with prior research [49], in which peer networks provide support and guidance for individuals seeking mental health resources through information sharing and fostering connections among individuals with similar experiences. In addition, our findings did not indicate a strong reliance on universities’ websites as a source of information for students. This finding is inconsistent with the study result [50] that university websites and social media enhance mental health support by broadening access to mental health resources as sources of information for mental health help. Students in this study may prefer and trust face-to-face communication over online resources. Thus, universities and counseling services need to consider implementing web-based focused support strategies to ensure that students utilize online resources efficiently.

This study revealed that students hold a positive mental help-seeking attitude, and their intentions to seek help are moderate. This result is consistent with a study by [51] among Turkish students. This similarity suggests that university students across different cultures may hold comparable positive attitudes toward seeking mental health support. This can be explained by the TPB, in which students’ intention to seek professional help is directly influenced by students’ attitudes and perceived behavioral control and indirectly influenced by factors such as subjective norms and self-stigma [52]. In our study, the overall mean for EI indicates a moderate level, which aligns with previous studies that assessed EL levels among nursing students [53, 54]. Highlighting that in Saudi Arabia, a significant proportion of students (96.6%) had moderate to high levels of EI [54]. In Saudi Arabia, nursing colleges focus on both academic and interpersonal skills. Students are trained to interact with diverse patients and nurses and communicate effectively in academic and clinical settings. Hence, this contributes to the development of EI among nursing students.

Our result from the multiple linear regression analysis identified several significant predictors of mental help-seeking intention. Particularly, EI was a significant positive predictor of professional psychological help-seeking intention, which is a novice finding. Although previous studies have shown that higher EI correlates with increased help-seeking behaviors. This might be explained as interpersonal emotional competence improves help-seeking intentions and indirectly supports educational success among students [55, 56]. In this study, it can be interpreted that students have emotional self-awareness and regulation, as well as interpersonal communication skills. This enables students to recognize their mental health needs and seek professional support.

Further, the mental help-seeking attitude had a strong positive effect on the mental help-seeking intention. This implies that attitudes toward help-seeking behaviors significantly predict intention to seek professional psychological help among nursing students. These findings are consistent with studies’ findings in various cultures [57, 58]. Another significant predictor was the participants’ history of consulting a mental health professional with the intention of seeking help. This finding is not aligned with [57], which showed university students with prior help-seeking experience were not influenced by their intention to seek help for depression [57]. This discrepancy can be explained by cultural perspectives toward mental health, which could shape how previous help-seeking experiences shape future intentions.

An interesting finding that students reported unavailability of on-campus counseling center and those were unsure about its availability had a significantly higher intention to seek professional psychological help. This aligns with a previous study [59, 60]. According to the study’s findings, age, gender, education level, university affiliation, and source of information about the on-campus counseling center were not significant predictors of intention to seek help from mental health professionals. This finding is inconsistent with [61], which might be attributed to cultural norms and the availability and effectiveness of services [62, 63]. Additionally, the source of information regarding counseling services is not a significant predictor of service utilization. This can be understood through the Saudi cultural lens, where mental health issues are viewed as private matters, with individuals’ preferences for solving psychological challenges within the family unit. Thus, students may rely more on family members and friends as informal sources of support rather than institutional sources. This reliance on informal support may influence students’ intentions to utilize on-campus counseling services.

In this study, EI is found to be a mediator between help-seeking attitudes and intentions. This suggests that students with EI are more likely to hold positive attitudes and intend to seek professional mental help. This could be explained by the EI’s role in the ability to appraise emotions positively, which can foster openness to seeking professional assistance [45]. In this study, students with EI were able to recognize and regulate their emotions and address their emotional distress through professional support. This positive appraisal might enhance their attitudes toward seeking psychological help [45, 64] and significantly predict their intentions to seek psychological counseling [65]. Additionally, the mediation effect of EI emphasizes the importance of integrating EI training into academic curricula, which could serve as preventive strategy.

### Implication to nursing

The findings of this study have important implications for nursing research, education, and practice. It fills gaps in the existing literature by shedding light on how emotional intelligence mediates the relationship between nursing students’ attitudes and intentions to seek professional psychological support in Saudi Arabia. This study contributes to nursing science through the application of the Theory of Planned Behavior (TPB) to explain how an individual’s intention to engage in a behavior and their perceived control over it affects their decision-making. In nursing education, it provides a strong justification for incorporating emotional intelligence into nursing curricula to enhance students’ mental health. Also, it informs policymakers in nursing colleges of the need to implement interventions and strategies to enhance nursing students’ attitudes and intentions to seek professional psychological support, which could be tailored to address nursing students in their second and third years. These implications can guide the development of interventions designed to cultivate a supportive and emotionally competent nursing workforce.

### Recommendation for future study

The study’s findings and limitations lead to several research recommendations. Replication of this study is recommended across different regions of Saudi Arabia to have a comprehensive understanding of study variables. Further, a longitudinal approach would be valuable in examining nursing students’ attitudes, intentions, and EI changes over time toward seeking professional psychological help. Furthermore, a qualitative approach is needed to get a more in-depth understanding of factors influencing nursing students’ intentions to seek professional psychological help within a cultural context.

### Limitation

This study has some limitations that warrant attention. The cross-sectional design of the study limits the causality relationship. However, the results provide observational support that EI mediates the effect between attitudes toward seeking professional psychological help and intention to seek such help. Further, the recruitment of students from one city in Saudi Arabia limits the generalizability of the results to all nursing students. Furthermore, the self-report measures used in the current study might have introduced response bias, in which students might have answered based on social expectations, which could have affected the validity of the study results. Future research might use different sampling methods to enable the interpretation of causality.

## Conclusion

The research found that moderate positive attitudes toward seeking professional psychological help and a moderate level of EI were associated with intentions to seek help among university students in Saudi Arabia. Based on these correlations, universities need to increase awareness programs that promote the benefits and availability of on-campus counseling services. Furthermore, universities need to implement targeted interventions to promote mental health education. Further, EI can be promoted through training programs that enhance students’ attitudes, such as emotional regulation and stress management skills. The findings can inform policymaking in nursing colleges, educators, and mental health professionals to develop culturally sensitive interventions and effective strategies to address mental health concerns and promote help-seeking intentions among nursing students.

## Electronic supplementary material

Below is the link to the electronic supplementary material.

Supplementary Material 1

## Data Availability

The data used in this study are available upon reasonable request from the corresponding author.

## Abbreviations

TPB: Theory of Planned Behaviour
EI: Emotional Intelligence
MHSIS: Mental Help Seeking Intention Scale
MHSAS: Mental Help Seeking Attitude Scale
WLEIS: Wong And Law Emotional Intelligence Scale
IQR: Interquartile Ranges
SEA: Self-Emotions Appraisal
OEA: Others-Emotions Appraisal
UOE: Use of Emotion
ROE: Regulation of Emotion
