# Supplementary material for: Nursing students’ attitudes and intentions towards seeking professional psychological help: the mediating role of emotional intelligence
Source: BMC Psychol. 2025 Feb 20;13:144. doi: 10.1186/s40359-025-02474-w (PMC11843813; doi:10.1186/s40359-025-02474-w)
Supplement: Supplementary file 1 — Supplementary Material 1 [file 40359_2025_2474_MOESM1_ESM.pdf]

Supplementary materials

Mental Help Seeking Attitudes Scale (MHSAS)

Please mark the circle that best represents your opinion. For example, if you feel that your seeking help would be extremely useless, you would mark the circle closest to "useless." If you are undecided, you would mark the "0" circle. If you feel that your seeking help would be slightly useful, you would mark the "1" circle that is closer to "useful."

If I had a mental health concern, seeking help from a mental health professional would be...

	3	2	1	0	1	2	3	
Useless	◯	◯	◯	◯	◯	◯	◯	Useful
Important	◯	◯	◯	◯	◯	◯	◯	Unimportant
Unhealthy	◯	◯	◯	◯	◯	◯	◯	Healthy
Ineffective	◯	◯	◯	◯	◯	◯	◯	Effective
Good	◯	◯	◯	◯	◯	◯	◯	Bad
Healing	◯	◯	◯	◯	◯	◯	◯	Hurting
Disempowering	◯	◯	◯	◯	◯	◯	◯	Empowering
Satisfying	◯	◯	◯	◯	◯	◯	◯	Unsatisfying
Desirable	◯	◯	◯	◯	◯	◯	◯	Undesirable

Mental Help Seeking Intention Scale (MHSIS)

INSTRUCTIONS: For the purposes of this survey, “mental health professionals” include psychologists, psychiatrists, clinical social workers, and counselors. Likewise, “mental health concerns” include issues ranging from personal difficulties (e.g., loss of a loved one) to mental illness (e.g., anxiety, depression). Please mark the box that best represents your opinion.

If I had a mental health concern, I would intend to seek help from a mental health professional.

1 (Extremely unlikely)	2	3	4	5	6	7 (Extremely likely)

If I had a mental health concern, I would try to seek help from a mental health professional.

1 (Definitely false)	2	3	4	5	6	7 (Definitely true)

If I had a mental health concern, I would plan to seek help from a mental health professional.

1 (Strongly disagree)	2	3	4	5	6	7 (Strongly agree)

Wong and Law Emotional Intelligence Scale (WLEIS)

Direction:

Responses can range from a 7-point Likert scale (1 = totally disagree, 2 = disagree, 3 = somewhat disagree, 4 = neither agree nor disagree, 5 = somewhat agree, 6 = agree, 7 = totally agree)

I)Self-Emotions Appraisal (SEA)

1.I have a good sense of why I have certain feelings most of the time.
2.I have good understanding of my own emotions.
3.I really understand what I feel.
4.I always know whether or not I am happy.

II) Others-Emotions Appraisal (OEA)

5.I always know my friends' emotions from their behavior.
6.I am a good observer of others' emotions.
7.I am sensitive to the feelings and emotions of others.
8.I have good understanding of the emotions of people around me.

III)Use of Emotion (UOE)

9. I always set goals for myself and then try my best to achieve them.
10. I always tell myself I am a competent person.
11. I am a self-motivating person.
12. I would always encourage myself to try my best.

VI)Regulation of Emotion (ROE)

13. I am able to control my temper so that I can handle difficulties rationally.
14. I am quite capable of controlling my own emotions.
15. I can always calm down quickly when I am very angry.
16. I have good control of my own emotions.

Permission to use measurements must be obtained from the original authors before use.
